# Trends in Antithrombotic Therapy Initiation Among Patients With Stroke Pre‐ and Post–COVID‐19 in Sweden—An Interrupted Time Series Study

**DOI:** 10.1111/bcpt.70213

**Published:** 2026-02-12

**Authors:** Salar Mousa, Katarina Persson, Maria Palmetun‐Ekbäck, Fredrik Nyberg, Huiqi Li, Björn Wettermark, MohammadHossein Hajiebrahimi

**Affiliations:** ^1^ School of Medical Sciences, Faculty of Medicine and Health Örebro University Örebro Sweden; ^2^ Pharmacology and Therapeutic Department, Faculty of Medicine and Health Örebro University Örebro Sweden; ^3^ School of Public Health and Community Medicine, Institute of Medicine, Sahlgrenska Academy University of Gothenburg Gothenburg Sweden; ^4^ Pharmacoepidemiology Unit, Department of Pharmacy Uppsala University Uppsala Sweden

**Keywords:** antithrombotic therapy, COVID‐19, drug utilization, stroke, Sweden

## Abstract

**Background:**

Antithrombotic therapy constitutes a critical part of stroke management, and its utilization serves as an important indicator in the assessment of stroke care. However, knowledge on how the pandemic influenced the antithrombotic utilization after stroke remains limited.

**Objective:**

This study aims to investigate the impact of the COVID‐19 pandemic on the initiation of antithrombotic agents after stroke in Sweden.

**Design:**

Using nationwide linked Swedish health registers, we conducted an interrupted time series analysis (ITSA) using generalized linear model (binomial distribution, logit link). We estimated monthly trends in the initiation of antithrombotic drugs among new stroke patients between January 2019 and April 2024, with the pandemic intervention point set in March 2020.

**Key Results:**

The monthly number of stroke cases declined significantly by 107 cases per month. No significant changes were observed in the total antithrombotic initiation. Antiplatelets showed a significant pre‐pandemic increasing trend (OR = 1.011 per month, 95% CI: 1.003 to 1.020), an immediate level increase at onset (OR = 1.150, 95% CI: 1.067 to 1.259) and a post‐intervention decline (OR = 0.991 per month, 95% CI: 0.981 to 0.998). Factor Xa inhibitors use increased post‐intervention (OR = 1.004, 95% CI: 1.002 to 1.006), while warfarin (OR = 0.990, 95% CI: 0.981 to 0.999) and dabigatran (OR = 0.991, 95% CI: 0.986 to 0.997) decreased.

**Conclusions:**

The COVID‐19 pandemic had no impact on the overall use of antithrombotic agents. However, a short‐term increase in antiplatelet initiation and long‐term changes in warfarin, dabigatran, and factor Xa initiations were observed after the COVID‐19 outbreak.

## Plain Language Summary

The thrombotic risk induced by COVID‐19 is studied worldwide. This study examined the pandemic’s effect on the initiation of antithrombotic medicines among patients with stroke in Sweden. Using nationwide Swedish health registers from 2019 to 2024, we found that stroke frequency was not influenced by the pandemic as hypothesized by other studies, and the overall use of antithrombotic medicines remained stable over time. However, temporary changes were observed in some of the medication classes in the early months of the pandemic.

## 1 Introduction

Stroke remains one of the major leading causes of mortality and disability worldwide, placing a large burden on society [1, 2, 3]. Different subtypes are presented in stroke diagnosis, with ischemic stroke being the most common [4, 5]. Although there has been improvement in primary prevention such as diet, physical activity, BMI control, avoiding tobacco and better treatment of hypertension and hyperlipidaemia, the burden of stroke has increased globally over the past three decades [2, 6, 7, 8]. However, the number of stroke cases in Sweden has declined substantially in the last decades [9]. Furthermore, a previous stroke or transient ischemic attack (TIA) is a well‐established risk factor for stroke recurrence, accounting for 20% of ischemic stroke cases in Sweden in 2023, highlighting the importance of secondary prevention, with the key component being antithrombotic therapy, but also complemented with antihypertensive therapy, statin therapy and lifestyle modification such as healthy diet and physical activity [4, 5, 10].

Following the outbreak of the COVID‐19 pandemic in March 2020, several studies reported increased stroke risk in infected patients both in acute and post‐acute phases. Stroke subtypes varied between large‐vessel occlusion, small‐vessel occlusion, haemorrhages and cerebral venous thrombosis, suggesting that inflammation‐related mechanisms such as immune dysregulation, cytokine storms and the subsequent endothelial dysfunction contributed to the pathogenesis [11, 12, 13, 14, 15, 16]. On the other hand, the pandemic's impact on society extended beyond individuals, affecting healthcare systems. Patients with chronic diseases—including cardiovascular conditions and neurological disorders—that require continuous access to healthcare and pharmaceuticals were particularly vulnerable due to increased infection risk and disruption in healthcare delivery [17].

Stroke care was early affected by the pandemic, with a noticeable decrease in the number of registered stroke cases compared with previous years [18, 19]. This change was likely due to shifts in healthcare prioritization, decreased access to healthcare generally and fear of acquiring COVID‐19 infection [20]. Given the vulnerable nature of patients with stroke—due to older age, several comorbidities, poor functional outcomes and decreased quality of life—such disruption in health‐care services might have posed significant challenges [21, 22, 23]. Although stroke care in Sweden was not affected to the same extent as in other settings [24], a recent study suggested an increased risk of developing stroke following COVID‐19 infection in the general Swedish population [25]. Moreover, increased mortality due to COVID‐19 may have acted as a competing risk for patients with stroke, as several studies have reported higher in‐hospital mortality rates among critically ill patients with COVID‐19 before secondary prevention could be initiated [26, 27].

Drug utilization patterns were also affected by the pandemic, both as a consequence of the impact of the disease and because of lockdowns and restrictions in access to healthcare and pharmacies. However, reports concerning drug utilization worldwide were heterogeneous. While some studies showed a temporary change in utilization of certain medications due to pandemic‐related policy measures [28, 29, 30], other studies suggested more persistent changes in drug utilization after the outbreak of the pandemic, indicating long‐term consequences of the pandemic on public health [31, 32, 33]. Despite the interplay between COVID‐19 and stroke and the challenges with access to care and medicines outlined above, limited information is available regarding the impact of the pandemic on the utilization of antithrombotics among patients with stroke. Therefore, this study aimed to investigate trends in the initiation of antithrombotic agents in patients with stroke in Sweden before and after the outbreak of the COVID‐19 pandemic.

## 2 Materials and Methods

The study was conducted in accordance with the Basic & Clinical Pharmacology & Toxicology policy for experimental and clinical studies [34].

### 2.1 Setting

This interrupted time series analysis (ITSA) was conducted in Sweden, which is well‐known for its tax‐funded healthcare system with complete coverage, national guidelines for stroke management, large population‐based registries, systematic quality control of stroke care and a nationwide network of stroke units [35, 36]. The study consisted of patients aged > 18 with a first (incident) specialist healthcare contact (hospitalization or specialist ambulatory care visit) for ischemic stroke (ICD‐10:I63) or transient cerebral ischemic attacks (TIA) (ICD‐10:G45) between Jan 2019 and Apr 2024. To identify new users of antithrombotic therapy, patients with any previous dispensations of antithrombotic agent from January 2018 until the date of the index diagnosis were excluded.

### 2.2 Data Source and Classification

Data came from the Swedish COVID‐19 Investigation for Future Insights—a Population Epidemiology Approach using Register Linkage (SCIFI‐PEARL) project [37]. The SCIFI‐PEARL project linked several national registers, including the National Patient Register (NPR) [38], Swedish Prescribed Drug Register (SPDR) [39], the national database of notifiable diseases (SmiNet) [40], the National Cause of Death Register (NCDR) [41], and the Longitudinal Integrated database for health insurance and labour market studies (LISA) [42]. All data were linked at the individual patient level using the Swedish national personal identification number, and subsequently pseudonymized before granted access for research [43]. In the current analysis, we used data on diagnoses from the NPR, data on dispensed prescription drugs from SPDR, and data on sociodemographic characteristics for all patients from LISA based on the latest available data (at the end of 2019). Variables were classified as follows: age groups (18–44, 45–64, 65–74, 75–85 and > 85), marital status (married/unmarried), country of birth (Sweden, Nordics excluding Sweden, EU28 except the Nordics or out of EU28), education level (primary school [≤ 9 years], secondary school [10–12] and academic level [≥ 12 years]), employment status (employed, unemployed). Missing values were reported for each category separately.

### 2.3 Statistical Methods

We investigated monthly trends of antithrombotic dispensation among patients with stroke before and after the outbreak of the COVID‐19 pandemic in March 2020. Antithrombotic drugs were identified using the following Anatomical Therapeutic Chemical (ATC) codes: (1) B01A (all antithrombotics); (2) B01AA (warfarin); (3) B01AB (heparins); (4) B01AC (antiplatelets); (5) B01AE (direct thrombin inhibitors); and (6) B01AF (factor Xa inhibitors). We used a generalized linear model (GLM) with a binomial distribution and logit link function to estimate changes in the monthly odds of antithrombotic use in patients with incident stroke. The dependent variable was to be dispensed an antithrombotic prescription. For each calendar month, the total number of incident stroke cases constituted the denominator, and the numerator comprised those patients who received antithrombotic dispensation during the same month. A 2‐month treatment capture period was applied to account for potential delay between stroke diagnosis and prescription drug treatment initiation to ensure accuracy in the linkage between incident stroke diagnosis and the corresponding initiating antithrombotic drug dispensation for each individual, for example due to prolonged hospitalization [44]. The model was specified as follows (see Text Box 1 for details): logit (p_t_) = β₀ + β₁(Time_t_) + β₂(Post_t_) + β₃(Time_t_ × Post_t_).

Text Box 1 **Logistic regression model specification and interpretation of the coefficients.**

• p_t_: probability of antithrombotic dispensation in month t.

• β_0_: baseline log‐odds of dispensation before the pandemic.

• β_1_: pre‐pandemic trend.

• β_2_: immediate change in level following the pandemic.

• β_3_: post‐pandemic trend.

• β_1_ + β_3_: post‐pandemic trend slope.

Patients were classified as either being dispensed or not (yes/no), and binomial logistic regression was used to model the binary outcome [45]. Model diagnostics supported the adequacy of the model. Dispersion ratio suggested no overdispersion and diagnostic plots for residuals showed no significant patterns, therefore indicating a good model fit (Figure S1). Autocorrelation was assessed using the Durbin–Watson test, which indicated no significant first‐order autocorrelation. Additionally, the Breusch–Godfrey, Ljung–Box test and autocorrelation function (ACF) (Figure S2) confirmed the absence of higher‐order and seasonal autocorrelation indicating that adding seasonal terms to the model were unnecessary. The primary outcome was the monthly odds of receiving a first antithrombotic dispensation after stroke diagnosis. All statistical analyses were performed using R Version 4.4.1.

### 2.4 Sensitivity Analysis

To assess the robustness of the findings in our paper, two sensitivity analyses were performed. First, the influence of all‐cause mortality on the monthly numbers of stroke cases in our population was assessed by adding monthly all‐cause mortality as a time‐varying covariate to the ITS model. Autocorrelation was handled by using a first‐order autoregressive model (AR1). Second, to account for population changes in age structure over time, monthly age‐standardized stroke rates were also assessed, using direct standardization with a fixed standard population defined as the Swedish population age distribution in 2019. An ITS model was fitted to the age‐standardized rates, and effect estimates were reported using β coefficients representing the absolute change on the age‐standardized stroke rate per month.

## 3 Results

For the period before the onset of the pandemic, between Jan 2019 and Feb 2020, we recorded 19 047 stroke cases in Sweden (mean 70.6 years, median 72 years, IQR 62–80). The pre‐pandemic median of monthly stroke cases was 1278 (IQR 1240–1296), with an average of 1276 stroke cases per month. After the outbreak of the pandemic (post‐COVID period), between March 2020 and April 2024, we recorded 57 462 stroke cases (mean 70.5 years, median 72 years, IQR 62–80). The post‐pandemic median of monthly stroke cases was 1171 (IQR 1112–1200), with an average of 1158 stroke cases per month, corresponding to a reduction of 107 cases after the pandemic outbreak.

Sex distribution of stroke cases was similar across the study periods (Table 1). Females on average were approximately 4 years older than males at stroke diagnosis. Among males, the mean age at diagnosis was 68.6 years in both the pre‐ and post‐COVID periods (median 70 years, IQR 61–77 vs. 60–78). In females, mean age at diagnosis likewise remained unchanged at 72.5 years during the pre‐ and post‐COVID periods (median 74 years, IQR 65–82 in both periods). Furthermore, mean age remained consistent between males and females across the different age categories and COVID‐19 periods (see Table S1 in Appendix).

**TABLE 1:** Characteristics of participants of the study (patients diagnosed with stroke) in Sweden between Jan 2019 and Apr 2024.

	Pre‐COVID		Post‐COVID		Overall	
	Male	Female	Male	Female	Male	Female
	(*N* = 9054)	(*N* = 9230)	(*N* = 29 617)	(*N* = 28 608)	(*N* = 38 671)	(*N* = 37 838)
Age at stroke diagnosis						
18–44	390 (4.3%)	325 (3.5%)	1309 (4.4%)	1019 (3.6%)	1699 (4.4%)	1344 (3.6%)
45–64	2724 (30.1%)	1921 (20.8%)	8978 (30.3%)	5879 (20.6%)	11 702 (30.3%)	7800 (20.6%)
65–74	2789 (30.8%)	2487 (26.9%)	8539 (28.8%)	7432 (26.0%)	11 328 (29.3%)	9919 (26.2%)
75–84	2403 (26.5%)	2935 (31.8%)	8657 (29.2%)	9909 (34.6%)	11 060 (28.6%)	12 844 (33.9%)
≥ 85	748 (8.3%)	1562 (16.9%)	2134 (7.2%)	4369 (15.3%)	2882 (7.5%)	5931 (15.7%)
Marital status						
Married	4918 (54.3%)	3764 (40.8%)	16 163 (54.6%)	12 731 (44.5%)	21 081 (54.5%)	16 495 (43.6%)
Unmarried	4128 (45.6%)	5462 (59.2%)	13 293 (44.9%)	15 785 (55.2%)	17 421 (45.0%)	21 247 (56.2%)
Missing	8 (0.1%)	4 (0.0%)	161 (0.5%)	92 (0.3%)	169 (0.4%)	96 (0.3%)
Country of birth						
Sweden	7773 (85.9%)	7905 (85.6%)	25 154 (84.9%)	24 312 (85.0%)	32 927 (85.1%)	32 217 (85.1%)
Nordics excluding Sweden	362 (4.0%)	521 (5.6%)	1173 (4.0%)	1522 (5.3%)	1535 (4.0%)	2043 (5.4%)
EU28 except the Nordics	291 (3.2%)	273 (3.0%)	945 (3.2%)	942 (3.3%)	1236 (3.2%)	1215 (3.2%)
Europe except EU28 and the Nordics	174 (1.9%)	190 (2.1%)	677 (2.3%)	532 (1.9%)	851 (2.2%)	722 (1.9%)
Out of Europe	454 (5.0%)	340 (3.7%)	1666 (5.6%)	1299 (4.5%)	2120 (5.5%)	1639 (4.3%)
Missing	0 (0%)	1 (0.0%)	2 (0.0%)	1 (0.0%)	2 (0.0%)	2 (0.0%)
Education level						
Primary education (< 9 years)	2461 (27.2%)	2609 (28.3%)	7462 (25.2%)	7150 (25.0%)	9923 (25.7%)	9759 (25.8%)
Secondary education (10–12 years)	3994 (44.1%)	3844 (41.6%)	13 079 (44.2%)	11 967 (41.8%)	17 073 (44.1%)	15 811 (41.8%)
Tertiary education (> 12 years)	2494 (27.5%)	2659 (28.8%)	8612 (29.1%)	9078 (31.7%)	11 106 (28.7%)	11 737 (31.0%)
Missing	105 (1.2%)	118 (1.3%)	464 (1.6%)	413 (1.4%)	569 (1.5%)	531 (1.4%)
Employment status						
Employed	2635 (29.1%)	1833 (19.9%)	9013 (30.4%)	5841 (20.4%)	11 648 (30.1%)	7674 (20.3%)
Unemployed	581 (6.4%)	501 (5.4%)	1559 (5.3%)	1287 (4.5%)	2140 (5.5%)	1788 (4.7%)
Retired	5835 (64.4%)	6895 (74.7%)	18 941 (64.0%)	21 431 (74.9%)	24 776 (64.1%)	28 326 (74.9%)
Missing	3 (0.0%)	1 (0.0%)	104 (0.4%)	49 (0.2%)	107 (0.3%)	50 (0.1%)

**FIGURE 1:**
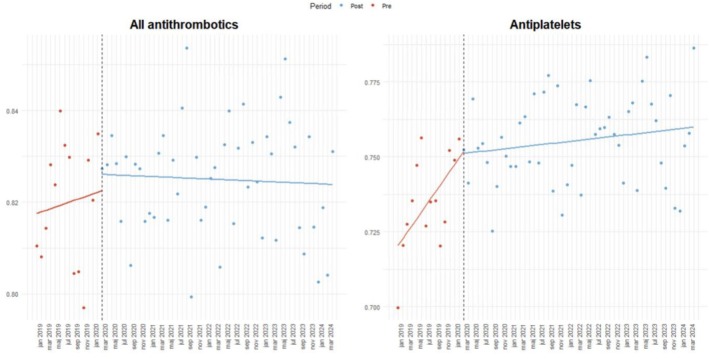
Interrupted time series analysis of the monthly proportion of patients with incident stroke initiating any antithrombotic and antiplatelet drug in Sweden, January 2019–April 2024. The x‐axis denotes time (months), and the y‐axis denotes the proportion of drug use per month. The vertical line indicates the onset of the COVID‐19 pandemic.

The pandemic did not significantly impact the total use of antithrombotic agents in patients with stroke (Figure 1). The proportion of patients using any antithrombotic agent at baseline was 81.7%. There was no statistically significant change in the trend prior to the pandemic (OR = 1.002, 95% CI: 0.993 to 1.012). The immediate level change in the initiation after the pandemic showed a non‐significant increase after the onset of the pandemic (OR = 1.066, 95% CI: 0.97 to 1.174). Post pandemic trend change showed a non‐significant decrease (OR = 0.997, 95% CI: 0.998 to 1.001). Overall, the onset of the pandemic was associated with both significant and non‐significant changes in the initiation of the different antithrombotic agents in Sweden, as presented in Table 2 below.

**TABLE 2:** Results from the interrupted time series analysis for the monthly proportions of patients with incident stroke initiating antithrombotic drug.

Drug class	Parameter	Coefficient	OR (95% CI)
All antithrombotic	β0 (%)	81.7	—
	β1	0.002	1.002 (0,993 to 1.012)
	β2	0.064	1.066 (0.97 to 1.174)
	β3	−0.003	0.997 (0.988 to 1.007)
	β1 + β3	−0.001	0.999 (0.998 to 1.001)
Antiplatelets	β0 (%)	71.9	—
	β1	0.011	1.011 (1.003 to 1.020)
	β2	0.150	1.150 (1.067 to 1.259)
	β3	−0.009	0.991 (0.981 to 0.998)
	β1 + β3	0.001	1.001 (1.00 to 1.002)
Factor Xa inhibitors	β0 (%)	7.37	—
	β1	−0.002	0.998 (0.984 to 1.012)
	β2	−0.084	0.916 (0.803 to 1.055)
	β3	0.006	1.006 (0.992 to 1.020)
	β1 + β3	0.004	1.004 (1.002 to 1.006)
Warfarin	β0 (%)	0.47	—
	β1	−0.031	0.969 (0.914 to 1.027)
	β2	0.105	1.105 (0.631 to 1.694)
	β3	0.022	1.022 (0.963 to 1.085)
	β1 + β3	−0.01	0.990 (0.981 to 0.999)
Heparin	β0 (%)	1.63	—
	β1	−0.014	0.986 (0.957 to 1.016)
	β2	0.138	1.138 (0.861 to 1.507)
	β3	0.014	1.014 (0.984 to 1.046)
	β1 + β3	0.001	1.001 (0.997 to 1.005)
Dabigatran (DTI)	β0 (%)	1.48	—
	β1	0.008	1.008 (0.979 to 1.038)
	β2	0.128	1.128 (0.835 to 1.537)
	β3	−0.016	0.984 (0.954 to 1.013)
	β1 + β3	−0.009	0.991 (0.986 to 0.997)

*Note:* β0: intercept value; β1: pre‐trend slope; β2: post‐interruption immediate level change; β3: post‐interruption trend change; β1 + β3: post‐interruption trend slope.

Subgroup analyses for the different drug classes showed that the antiplatelet utilization changed markedly. Prior to the pandemic, there was a significant upward trend in the probability of being dispensed antiplatelets after ischemic stroke (OR = 1.011, 95% CI: 1.003 to 1.020). At the intervention point at the outbreak of the pandemic, an immediate and significant level increase in the dispensations was observed (OR = 1.150, 95% CI: 1.067 to 1.259). However, this was followed by a significant negative trend in the months after (OR = 0.991, 95% CI: 0.981 to 0.998), indicating a slowdown in the monthly increase after the pandemic outbreak. As a result, the post pandemic trend (β₁ + β₃) showed a plateau‐like trend with a non‐significant change in the monthly odds (OR = 1.001, 95% CI: 1.00 to 1.002) (Table 2 and Figure 1).

**FIGURE 2:**
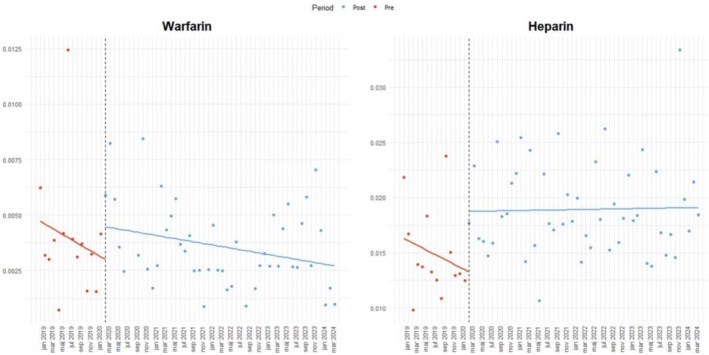
Interrupted time series analysis of the monthly proportion of patients with incident stroke initiating Warfarin and Heparin in Sweden, January 2019–April 2024. The x‐axis denotes time (months), and the y‐axis denotes the proportion of drug use per month. The vertical line indicates the onset of the COVID‐19 pandemic.

Furthermore, the use of warfarin showed non‐significant changes in pre trend (OR = 0.969, 95% CI: 0.914 to 1.027), immediate level change (OR = 1.105, 95% CI: 0.631 to 1.694) nor post‐trend change (OR = 1.022, 95% CI: 0.963 to 1.085). However, the post‐pandemic trend (β₁ + β₃) was borderline significant (OR = 0.990, 95% CI: 0.981 to 0.999), suggesting a possible decline in use (Figure 2). While for low molecular weight heparin utilization among stroke patients, neither the pre‐, post‐pandemic trends, nor the level change, reached significance (Table 2 and Figure 2).

**FIGURE 3:**
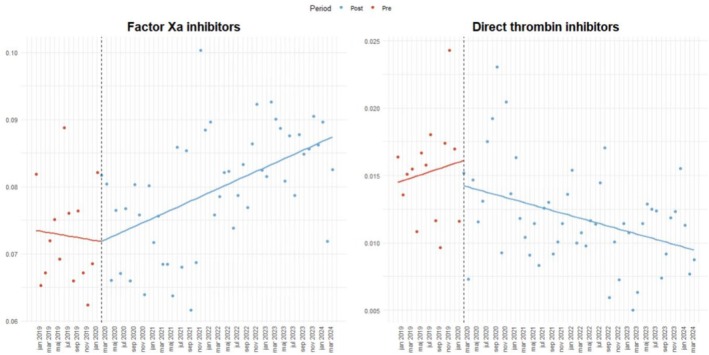
Interrupted time series analysis of the monthly proportion of patients with incident stroke initiating FXa inhibitors and DTI in Sweden, January 2019–April 2024. The x‐axis denotes time (months), and the y‐axis denotes the proportion of drug use per month. The vertical line indicates the onset of the COVID‐19 pandemic.

In contrast, the utilization of factor Xa inhibitors showed non‐significant decrease in the monthly odds prior to the pandemic (OR = 0.998, 95% CI: 0.984 to 1.012). While no immediate level change was observed (OR = 0.916, 95% CI: 0.803 to 1.055) nor a significant post‐pandemic change in the trend (OR = 1.006, 95% CI: 0.992 to 1.020), the monthly post‐pandemic trend (β₁ + β₃) was significantly positive (OR = 1.004, 95% CI: 1.002 to 1.006), indicating a delayed but steady rise in the use of factor Xa inhibitors following the outbreak (Figure 3). Furthermore, direct thrombin inhibitors (DTIs)—represented exclusively by dabigatran in this population—showed no significant pre‐, post‐ nor immediate level change. However, there was a significant decrease in the post‐pandemic trend (β₁ + β₃) (OR = 0.991, 95% CI: 0.986 to 0.997) indicating a decline in monthly dabigatran use on the long run after the outbreak of the pandemic (Table 2 and Figure 3).

After the onset of the pandemic, there was an immediate reduction in the monthly stroke cases (post‐intervention level change by −74 cases/month; *p* = 0.02), with no evidence of change in the post‐intervention slope. After including all‐cause mortality as a time‐varying covariate, the estimated change remained unchanged (−74 cases/month; *p* = 0.03), and the mortality term was not statistically associated with stroke counts.

In the sensitivity analysis using age‐standardized monthly stroke rates, no statistically significant immediate change (β = −5.3 × 10^−6^, corresponding to approximately −0.53 strokes per 100 000 population per month; *p* = 0.17) nor post‐intervention changes (β = 2.0 × 10^−7^, corresponding to approximately 0.2 strokes per 100 000 population per month; *p* = 0.58) were observed after the onset of the pandemic. Age‐standardized stroke rates (standardized to the Swedish population in 2019) remained stable over the study period.

## 4 Discussion

In this register‐based study, we found no statistically significant effect of the pandemic outbreak in March 2020 on the total antithrombotic use among patients with stroke in Sweden. However, subgroup analysis revealed a temporary increase in the initiation of antiplatelet agents immediately following the onset of the pandemic, which was subsequently followed by a stabilization of the trend, suggesting an adjustment to the initial response. In contrast, we observed a long‐term steady increase in the use of factors Xa inhibitors, alongside a long‐term sustained decline in the use of warfarin and dabigatran after the outbreak of the pandemic.

Crude stroke counts, on the other hand, decreased after the pandemic onset. However, sensitivity analysis using age‐standardized stroke rates and models adjusted for monthly all‐cause mortality did not show statistically significant changes, suggesting that the observed reduction may reflect the continuation of the pre‐existing downward trend of stroke incidence in Sweden rather than a pandemic‐specific change in stroke risk in different age‐groups [9, 46].

During the early days of the pandemic, the safety and efficacy of antithrombotic therapy among critically ill patients were assessed, with researchers aiming to explore the risk–benefit outcome of antithrombotic therapy on the overall COVID‐19–related mortality [47, 48]. These findings contributed to the emergence of new clinical guidelines recommending the use of antithrombotic therapy in critically ill and hospitalized patients as thromboprophylaxis measures [49].

These new guidelines were implemented in Sweden during the early phases of the COVID‐19 pandemic [50]. The prophylaxis measures applied in Sweden have possibly attenuated the numbers of stroke cases in Sweden, which aligns with our findings showing no significant change in overall antithrombotic initiation and a decline in the overall stroke cases across the time series, despite the thrombosis hypothesis suggesting an increase in the risk of developing stroke following COVID‐19 infection [12, 19, 25]. Similar results were obtained in a cross‐national comparative drug utilization study, which showed no overall change in the overall drug consumption in Sweden, except for a slight reduction in the prescription volume of antithrombotic agents, expressed as DDD/TID, by 0.8% in the first year of the COVID‐19 pandemic compared with the preceding year [51]. Moreover, competing mortality may explain the observed decline in the number of stroke cases despite the hypothesized increased risk of stroke associated with the COVID‐19 infection. Several studies on patients with stroke have shown higher in‐hospital mortality rates in critically ill patients with COVID‐19, affecting the numbers of incident stroke cases [26, 27]. Additionally, a Swedish study examined the national mortality patterns during the pandemic and showed a shift in mortality trends across several disease categories [17]. These studies highlight that the pandemic's effect on mortality patterns is critical and should be considered when interpreting stroke incidence and treatment patterns.

The initial change in the initiation of antiplatelets among patients with ischemic stroke may illustrate a possible short‐term behavioural change after the outbreak of the pandemic, potentially caused by the restrictive measures in Sweden during the early days of the pandemic, which relied on voluntary actions from individuals, leading to stockpiling behaviour among patients [52, 53, 54]. Another explanation could be that COVID‐19 improved medication adherence; studies from Italy and Canada have shown an increased cardiovascular medication adherence in both incident and prevalent users, possibly due to healthcare system adaptation and factors related to patients characteristics [55, 56].

Additionally, our study showed a significant long‐term increase in the initiation of factor Xa inhibitors, accompanied by a decline in warfarin use after the pandemic. This aligns with previous Swedish reports where guidelines suggest higher priority for treatment with direct oral anticoagulants (DOAC) compared with warfarin, and initiations of DOAC increased from 79% to 85% between 2018 and 2024 in patients with ischemic stroke [5]. However, a recent study by Samulesen et al. reported no significant change in the incidence of anticoagulant use among patients with atrial fibrillation in Sweden, with no major impact from the pandemic on the long run [57]. This discrepancy could be attributed to differences in study populations and outcome measures. Notably, Samuelsen et al. found similar results regarding warfarin use in Sweden, suggesting an underlying shift in prescribing preferences from warfarin to DOAC in several therapeutic areas may have been accelerated by the pandemic outbreak [57, 58]. Dabigatran use, on the other hand, decreased post‐pandemic onset in the long run, likely due to its less favourable pharmacokinetic and safety profile compared with other DOACs [59].

Globally, reduced healthcare access during early lockdowns negatively affected chronic cardiovascular care [60, 61, 62, 63]. However, our findings suggest that ischemic stroke management in Sweden was not affected by the pandemic to the same degree, likely due to effective thromboprophylaxis measures implemented during the early days of the pandemic.

This population‐based, nationwide study has several strengths. We used comprehensive data from multiple national registers, with complete and valid data on dispensed prescriptions from all healthcare providers in primary and secondary care. Furthermore, since the demographic profile in our study is similar to the national and international audit [64, 65], it enhances the generalizability of our findings. Moreover, using incidence measures with a washout period provides more information on the dynamics of the impact of the intervention compared with prevalence or aggregated counts of prescriptions.

Nonetheless, several potential limitations of this study should be acknowledged. Diagnoses were obtained from the National Patient Register (NPR), which, despite its extensive coverage, may include false positives for acute stroke [66]. Furthermore, policy shifts during the pandemic were not accounted for, introducing potential time‐dependent confounding, which ITSA methodology assumes to be absent [67, 68]. The short pre‐intervention period (1 year) limited the ability to fully capture and assess seasonal patterns, despite conducting several models to check for the seasonal factor in the trend analysis. Finally, the construction of the incidence measure was done by excluding patients with previous dispensation of the studied drugs, which remains a potential source of misclassification.

## 5 Conclusion

The COVID‐19 pandemic has posed significant global challenges on the management of non‐communicable diseases. However, our study found no statistically significant changes in the overall initiation of antithrombotic agents in patients with stroke in Sweden before and after the pandemic. While a short‐term increase in the initiation of antiplatelet agents among our population was observed at the onset of the pandemic, this trend later stabilized, suggesting a short‐term response to the pandemic.

## Author Contributions

Salar Mousa conceived and designed the study, collected data, conducted the statistical analyses, interpreted the results and wrote the initial manuscript. Mohammadhossein Hajiebrahimi contributed to the study design, data collection and analysis and interpretation of the results. Katarina Persson and Maria Palmetun‐Ekbäck contributed substantially to study design, providing critical input on data interpretation, manuscript writing as well as revision of important intellectual content. Björn Wettermark and Fredrik Nyberg were key contributors in the study design, statistical method, data interpretation and critical revision of the manuscript. Huiqi Li contributed to data collection and provided guidance in the statistical analysis. All authors contributed to the manuscript writing, reviewed the final draft and approved the final version for publication.

## Funding

This work was supported by Hjärt‐Lungfonden, 20210030, 20210581, 20240726; Forskningsrådet om Hälsa, Arbetsliv och Välfärd, 2024‐01711; Svenska Forskningsrådet Formas, 2020‐02828.

## Ethics Statement

This study is part of the SCIFI‐PEARL project which has been approved by the Swedish Ethical Review Authority, approval number [2020‐01800, 2020‐05829, 2021‐00267, 2021‐00829, 2021‐02106, 2021‐04098] and was conducted in accordance with the ethical standards of the 1964 Declaration of Helsinki and later comparable ethical standards.

## Conflicts of Interest

Fredrik Nyberg, Huiqi Li report participation in research projects funded by pharmaceutical companies (regulator‐mandated phase IV and investigator‐initiated studies), paid to the University of Gothenburg where they are employed (no personal fees), and with no relation to the work reported here. Fredrik Nyberg holds some AstraZeneca shares.

Salar Mousa, Björn Wettermark, MohammadHossein Hajiebrahimi, Katarina Persson, and Maria Palmetun‐Ekbäck declare no conflicts of interest.

## Data Availability

The data that support the findings of this study are not openly available due to reasons of sensitivity and are available from the corresponding author upon reasonable request. Data are located in controlled access data storage at Gothenburg University https://doi.org/10.2147/CLEP.S312742. The R scripts used for data analysis in this study are available from the corresponding author upon reasonable request.
